# Volatile Cues from Fresh Cattle Dung Can Drive Horn Fly Egg-Laying and Fecal Attraction to Horn Flies, *Haematobia irritans* (Diptera: Muscidae)

**DOI:** 10.3390/insects16020129

**Published:** 2025-01-28

**Authors:** Javier Espinoza, Isabel Soto, Joaquín Arriagada, Marcelo Lizama, Nehuen Aninao, Washington Aniñir, Emilio M. Ungerfeld, Manuel Chacón-Fuentes, Andrés Quiroz

**Affiliations:** 1 Laboratorio de Ecología Química, Departamento de Ciencias Químicas y Recursos Naturales, Universidad de La Frontera, Casilla 54-D, Avenida Francisco Salazar 01145, Temuco 4811230, Chile; i.soto03@ufromail.cl (I.S.); j.arriagada@ufromail.cl (J.A.); m.lizama04@ufromail.cl (M.L.); n.salazar13@ufromail.cl (N.A.); w.aninir01@ufromail.cl (W.A.); andres.quiroz@ufrontera.cl (A.Q.); 2 Centro de Excelencia en Investigación Biotecnológica Aplicada al Medio Ambiente (CIBAMA), Universidad de La Frontera, Avenida Francisco Salazar 01145, Temuco 4811230, Chile; 3 Programa de Doctorado en Ciencias de Recursos Naturales, Universidad de La Frontera, Av. Francisco Salazar 01145, Temuco 4811230, Chile; 4 Programa de Doctorado en Ciencias Agroalimentarias y Medioambiente, Facultad de Ciencias Agropecuarias y Medioambiente, Universidad de La Frontera, Temuco 4811230, Chile; 5 Facultad de Ingeniería y Ciencias, Universidad de La Frontera, Av. Francisco Salazar 01145, Temuco 4811230, Chile; 6 Centro Regional de Investigación Carillanca, Vilcún, Instituto de Investigaciones Agropecuarias (INIA), Región de La Araucanía, Temuco 7500502, Chile; emilio.ungerfeld@inia.cl; 7 Agriaquaculture Nutritional Genomic Center, CGNA, Temuco 4780000, Chile; manuel.chacon@cgna.cl

**Keywords:** fecal volatile compounds, semiochemicals, cattle flies, olfactory response, oviposition

## Abstract

The horn fly is an economically important hematophagous ectoparasite of cattle. Its management relies heavily on broad-spectrum pesticides, which are harmful to the environment and have led to the development of resistance. Therefore, alternative control methods are needed. Semiochemicals involved in communication between horn flies and their host are a promising alternative. Considering that the egg-laying of this fly occurs almost exclusively in fresh cattle dung, and most parts of its life cycle occur totally in dung, dung volatiles might play an important role in horn fly behavior. Therefore, the main aim of this study was to evaluate the effects of volatile blends and compounds emitted from fresh/aged cattle dung on the olfactory response and oviposition of horn flies. Dung blends were captured and analyzed by SPME-GC/MS. *p*-Cresol was the most abundant compound in dung blends, followed by *α*- and *β*-pinene, limonene, and *β*-caryophyllene, among other common dung volatiles. Fresh-dung volatiles attracted males and females in a Y-tube olfactometer, and they elicited the egg-laying of flies in two-choice tests. *p*-Cresol and α-pinene were attractive to females and they elicited higher oviposition, demonstrating that dung volatile semiochemicals, in part, lend to dung attractiveness and stimulate the horn fly oviposition.

## Simple Summary

The horn fly is one of the most important ectoparasites of cattle worldwide. Insecticides are commonly used to mitigate this plague, thought resistance to conventional insecticides and its environmental impact are problematic; hence, new approaches for management are being investigated. The use of chemical cues involved in horn fly behavior is promising because these cues are naturally and specifically detected by horn flies, avoiding the development of resistance. In this work, the effects of volatile blends and compounds emitted from cattle dung on the olfactory response and oviposition of horn flies were evaluated. The olfactory response and the egg-laying of horn flies were influenced by volatile cues emanated from cattle feces. Volatile cues from fresh cattle dung were preferred by flies of both sexes and by flies for egg-laying. Moreover, females were attracted to *p*-cresol and *α*-pinene which elicited a higher oviposition. Therefore, *p*-cresol and *α*-pinene may be responsible, in part, for the dung selection by female horn flies searching for oviposition sites. These results can be valuable for the development of new approaches for the control of the horn fly using semiochemicals as lures in fly traps and simultaneously emitting repellents from cattle as part of a “push-pull” strategy.

## 1. Introduction

The horn fly, *Haematobia irritans irritans* (L.) (Diptera: Muscidae), is an obligate hematophagous ectoparasite of cattle, considered one of the most economically important fly pests of livestock production worldwide [1,2,3]. It is a little brown-gray fly between 3 and 5 mm in length, which makes it the smallest biting fly parasitizing beef cattle [1,4,5,6,7]. *H. irritans* is widely distributed in many of the temperate, tropical, and subtropical regions of the Northern Hemisphere, including Europe, Asia Minor, and North Africa [1]. In the Americas, *H. irritans* ranges from southern Canada to temperate areas of Argentina, Uruguay [1,8,9,10,11], and Chile [11]. In Chile, the species was definitively established in 1993 [11,12] and nowadays is found between the regions of Arica y Parinacota and Aysén [12].

The horn fly is holometabolic through four successive stages: egg, larva, pupa, and adult fly [1,5]. The flies spend most of their adult life on the cattle host, feeding and mating only on the host [13,14]. One day after copulation, females leave the host for egg-laying, just when the host defecates [15]. Then, the eggs are almost exclusively laid on fresh dung [16]. It has been reported that dung ceases to be attractive to flies a few minutes of being dropped [14,15]. During its lifetime, a single female can produce 78 to 400 eggs deposited in clusters of 20–25 eggs [1,17]. Hatching occurs 1-2 days after oviposition and the larvae reside within the dung, passing through three instars. The final instar initiates pupariation inside of the dung pat or around the dung pat [1]. Development from egg to adult has been reported at 7–11 [18] and 9–12 days [19], depending on the temperature [20]. After hatching, flies choose an animal host, and they feed off it for the first time. The adult lifetime is between 6 and 8 weeks [21]. It has been estimated that the whole lifecycle of the *H. irritans* takes 10–20 days when environmental temperature fluctuates between 20 and 30 °C in places with high levels of humidity [5]. In Chile, flies emerge from November to May, showing a large population from December to March [12]. However, because of climate change, seasonality has decreased, and the season has been extended [13].

Both female and male horn flies use their piercing proboscis [1,22] to feed on cattle multiple times per day [23,24]. Fly feeding behavior causes extreme annoyance and stress to cattle, which waste energy in removing flies, principally through head and tail tossing, trembling, and bunching and seeking refuge [25,26]. It leads to reduced sleeping and grazing time, a worsening of feed conversion efficiency, reduced milk production, and weight gain, especially when the animals are under a massive infestation (>200 horn flies per animal) [23,25,27,28,29]. Moreover, horn flies are important vectors in the development of mastitis by *Staphylococcus aureus* and teat atresia [1,30,31,32,33], and they are also an intermediate host of *Stephanofilaria stilesi*, a filarioid nematode that causes skin lesions on cattle [6,34]. Skin damage includes scarring and fiber separation, which leads to reduced leather quality and lower commercial value [35].

In the US livestock industry, the monetary damages caused by *H. irritans* were estimated to be USD 876 million annually [36], which, when adjusted for inflation to 2021 dollars, is USD 1.75 billion per year [23,24]. In Mexico, estimated annual losses amount to USD 231.66 million [37], whereas annual losses in Brazil were estimated to be from USD 150 million to USD 2.56 billion in 2012 [38]. In Chile, the annual economic losses caused by the horn fly were valuated at CLP 25,800 million [39,40], equivalent to USD 45 million in January 2001 [41]. This estimation did not consider losses produced by reduced reproductive efficiency and leather quality [42].

Nowadays, *H. irritans* management is largely centered on conventional pesticides, mainly organophosphates and pyrethroids commercialized in the form of ear-tags, pour-on, sprays, and others. Regrettably, pesticides are harmful to the environment and highly persistent, they have significant residual toxicity, and they have led to the development of resistant horn fly populations [43,44,45]. Hence, alternative control strategies are really necessary [1,22]. Other control methods such as the monitoring of horn fly loads [46,47], pasture management [48], the use of physical trapping [49], horn fly-resistant cattle breeds [29,50,51,52], natural predators [35,53,54], coprophagous beetles [55,56,57], entomopathogenic fungi [56,57,58,59,60,61,62], plant-derived natural products [15,21,30,63,64,65,66,67,68,69,70,71,72,73,74], grasses infected with endophytic fungi [75,76] and their isolated active compounds [41,77,78], and novel vaccines [35] have been investigated as alternative strategies. However, the field implementation of those methods has, until now, been very limited.

The use of semiochemicals, either volatile or non-volatile, mediating the horn fly intra- and inter-specific communication and host selection [21,35,79,80,81,82,83,84] is one of the most promising approaches because they use compounds naturally detected by specialized olfactory receptor neurons located either on the antennae or on the maxillary palps of insects [80,81,82], avoiding the development of insecticide resistance.

Biting and blood-sucking flies, including cattle flies, use a variety of long- and short-range visual, olfactory, gustatory, and physical cues in host location and selection. Among olfactory stimuli, volatile semiochemicals play an important role in mediating such behavior [71,72]. It has been demonstrated that individual animals within herds vary in their attractiveness for *H. irritans* flies [85,86]. Moreover, the natural differential attractiveness within herds for cattle flies, including horn flies [21], is partly due to differences in volatile semiochemicals emitted from the host [87].

Many studies have identified semiochemicals involved in host location and selection for tsetse flies [88,89,90,91,92,93]. It also has been demonstrated that the stable fly, *Stomoxys calcitrans* (L.) (Diptera: Muscidae), phylogenetically related to the horn fly [82], is able to discriminate among host cattle of different physiological statuses [93]. However, the behavioral and electrophysiological responses of the stable fly to the host odor have focused on compounds established as semiochemicals for tsetse flies [94,95,96]. For other cattle flies, including horn flies, little is known of the chemical nature and role of semiochemicals used in host location and selection.

Considering that the egg-laying of female horn flies occurs almost exclusively in fresh cattle dung [17], and three of the four stages of the horn fly life cycle occur exclusively in cattle dung, volatile semiochemicals emitted from cattle dung might play an important role in mediating the dung selection and oviposition of the horn flies. To date, there is related information in the literature for *S. calcitrans*. It has been reported that horse and cow manure were attractants to stable flies, especially females searching for oviposition sites [97,98]. The major EAG active compounds in both substrates, were butanoic acid, 1-octen-3-ol, decanal, octan-3-one, *p*-cresol, skatole, *β*-caryophyllene, and dimethyl trisulfide [97]. Behavioral responses of stable flies to volatile compounds emanating from manure slurry (mixture of cattle manure, urine, water, and accumulated organic waste) have also been investigated [99]. Methoxyphenyl oxime, phenol, *m*-cresol, *p*-cresol, and 4-ethylphenol were detected by GC-MS of solid-phase microextraction (SPME) captures from cattle manure slurry. Olfactory bioassays showed that phenol, *p*-cresol, and *m*-cresol at the lowest dosages tested elicited significant attraction, but at higher dosages, they appeared to be unattractive, even acting as repellents for stable flies. Moreover, mixtures of phenol and either *m*-cresol or *p*-cresol at lower concentrations were attractive to stable flies. Additionally, traps placed in fields with an application of manure slurry caught over 10 times more flies than those placed away from sites sprayed with slurry, and traps baited with lures containing blends of phenol and *m*-cresol or *p*-cresol captured more stable flies compared with the control [99]. Notwithstanding the aforementioned, nothing is known about the role of volatile semiochemicals emitted from cattle dung in dung selection and the oviposition of the horn fly. Thus, the main aim of this study was to evaluate the effects of volatile blends and individual compounds emitted from fresh and aged cattle dung on the olfactory response of horn flies and on the oviposition of female horn flies.

## 2. Materials and Methods

### 2.1. Collection of Horn Flies and Sexing

Laboratory bioassays were conducted using wild *H. irritans* of unknown age, collected from beef cattle according to Espinoza et al. [41]. Thirty Holstein–Freisian cows were placed in a corridor at Maquehue-UFRO, Freire, Chile (38°83′ SL, 72°69′ WL, 200 masl). Then, horn flies on cows were hand-collected and caught in 1 L glass flasks (200–400 flies per flask). Flasks were subsequently sealed with an entomological net and were transported to the laboratory. Once in the laboratory, the horn flies were pooled together and maintained at 25 ± 2 °C with variable humidity (30–50%) under a 12:12 h light:dark photoperiod. Horn flies were starved and acclimatized for 24 h prior to bioassays.

To sex each horn fly used in bioassays, flies were individually maintained in 2 mL microcentrifuge tubes with 1.5 mL of Kahle’s solution after each bioassay. Each fly was immobilized in a freezer and immediately after, it was observed under stereo microscope (Model StereoBlue SB.1903, Euromex Microscopen b.v., Duiven, The Netherlands) to check the apex between the compound eyes. According to Brewer et al. [1], the male compound eyes have a single vertex with a single row of bristles on opposite sides of the midline of the eyes, and the female has a wider vertex between the compound eyes.

### 2.2. Collection of Cattle Feces

Six Holstein–Freisian cows having similar weight, height, color, and hair density were selected from the herd, and they were individualized by their ear tag numbers. These cows were kept in a sleeve, and then, fecal samples were obtained by anal stimulation. Feces were collected in sterilized bags, which were immediately transported to the laboratory. Each fecal sample was portioned into 4 sub-samples, and arranged by time after excretion. A first sub-sample of each animal was categorized as “fresh dung or 1-day old dung”, and it was used in bioassays during the first 24 h after excretion. A second sub-sample of each animal was categorized as “3-day old dung”, and it was kept under field conditions since excretion up to a limit of 72 h after. These sub-samples were used in bioassays during the 3rd day after excretion (48 h up to 72 h since excretion). A third sub-sample of each animal was categorized as “5-day old dung”, and it was kept under field conditions since excretion up to a limit of 120 h after. These sub-samples were used in bioassays during the 5th day after excretion (96 h up to 120 h since excretion). A fourth sub-sample of each animal was categorized as “7-day old dung”, and it was kept under field conditions since excretion up to a limit of 168 h after. These sub-samples were used in bioassays during the 7th day after excretion (144 h up to 196 h since excretion). To perform the olfactory and oviposition tests, the fecal sub-samples belonging to the same category were pulled together and homogenized to ensure the uniformity in bioassays.

### 2.3. Olfactometer Bioassays

A dual-port Y-tube glass olfactometer (stem, 110 mm; ports, 90 mm at a 130° angle from the stem; internal diameter, 10 mm) was used to assess the olfactory response of horn flies to dung volatiles, according to Espinoza et al. [41], with modifications.

The following compounds tested here were purchased from Sigma-Aldrich (St. Louis, MO, USA): *α*-pinene (≥97.5% purity (GC); CAS N°: 80-56-8; product number: 147524), *β*-caryophyllene (≥98.0% purity (GC); CAS N°: 87-44-5; product number: 22075), *D*-limonene (≥96.5% purity (GC); Enantiomeric excess (e.e.) ≥ 97.5%; CAS N°: 5989-27-5; product number: 183164), and *p*-cresol (≥98.5% purity (GC); CAS N°: 106-44-5; product number: C85751). *β*-pinene (≥95.0% purity (GC); CAS N°: 127-91-3; Product number: PHL89335) was purchased from PhytoLab GmbH & Co. KG (Vestenbergsgreuth, Deutschland).

A piece of filter paper (0.5 cm × 5 cm) was individually soaked on 10 μL of the following samples: (a) Fresh and aged cattle dung; (b) *D*-limonene, *α*-pinene, *β*-pinene, *β*-caryophyllene, and *p*-cresol at 0.05 μg μL^−1^ in *n*-hexane. For blanks, filter papers were soaked on 10 μL of *n*-hexane. These pieces of filter paper were placed in the middle of Pasteur pipettes, and each port of the Y-tube was connected to these pipettes, containing either sample or blank. The olfactometer was horizontally put over a white LED flat panel light, and it was connected from the base of the stem to a vacuum pump generating a purified air flow (200 mL min^−1^) to carry the volatile stimuli from the ports to the stem. The air was purified through activated carbon.

To test the horn fly responses to samples during the photophase, 24 h-starved male and female flies of unknown age were individually released into the olfactometer through a hole at 2 cm of the base of the stem and immediately afterward sealed in with a Teflon cap. Three minutes were given to each fly to choose between treatment or blank, and its preference in the treatment or blank port (>half of the port) was recorded. Olfactory responses were recorded as the percentage of flies inside the treatment or blank ports. Non-responding flies were not considered for the purpose of this calculation. Each sample was tested with 10 different flies per sex, and the bioassay was replicated 6 times until 60 flies per sample were evaluated.

The order of the Y-tube ports was reversed, and the olfactometer was rinsed and air-dried after two flies were tested. After the homogeneity of variance and normality of the data were confirmed, olfactory responses of flies among treatments were analyzed using parametric Student *t*-test (* *p* ≤ 0.05, ** *p* ≤ 0.01, *** *p* ≤ 0.001), followed by a Tukey test using the statistics program StatsDirect 3.1 (StatsDirect Ltd., Cheshire, UK).

### 2.4. Egg-Laying Bioassays

#### 2.4.1. Using Fresh and Aged Cattle Dung

Four PTE plastic jars were placed inside a screen cage (30 cm in depth × 20 cm in width × 10 cm in height); two of them contained 2 g of fresh or aged dung as treatment, and the other two were empty as blanks. Each jar was covered with a piece of white tulle (10 cm × 10 cm) to avoid the contact between flies and dung but allowing the volatiles to release from the dung. Blank and treatment jars were placed on opposite sides of the screen cage in a crossover model. Their positions were reversed among replications. Approximately 200 mixed-sex horn flies of unknown age, starved 24 h before testing, were put inside the screen cage in a female:male ratio of 6:4, approximately, and the cage was covered with a perforated lid. Four hours in darkness were given to the flies for egg-laying to avoid phototaxic behaviors. Immediately after, the cages were frozen, and the flies were removed from the cages. The number of eggs on the treatments and the blanks was counted. The number of females in each screen cage was also recorded. The egg-laying on the treatments or on the blanks is expressed as number of eggs laid per female. This bioassay was replicated six times per sample (day 1 fresh sample, day 3, day 5, and day 7). Differences in the number of eggs laid per female among treatments and blanks were analyzed using a generalized linear model (GLM) with a Poisson distribution and a log-link function, which is appropriate for count data. The analysis was performed using the statistical software Statistix 10 (Tallahassee, FL, USA). Model significance was assessed using the Likelihood Ratio Test. Post hoc comparisons between treatments were conducted by estimating predicted means and their confidence intervals, with non-overlapping intervals indicating significant differences.

#### 2.4.2. Using Aged Cattle Dung Amended with Fecal Volatile Compounds

Four PTE plastic jars were placed inside a screen cage (30 cm in depth × 20 cm in width × 10 cm in height); two of them contained 2 g of aged dung sample supplemented with individual volatile compounds, as treatment, and the other two contained 2 g of aged dung amended with 10 μL of *n*-hexane, as blank. Dung was supplemented with 10 μL of (a) *D*-Limonene, *α*-pinene, *β*-pinene, *β*-caryophyllene, and *p*-cresol at 0.05 μg μL^−1^ in *n*-hexane. The specifications of the compounds tested here were detailed in Section 2.3. Each jar was covered with a piece of white tulle (10 cm × 10 cm) to avoid contact between flies and dung but allowing the volatiles releasing from dung. Blank and treatment jars were placed on opposite sides of the screen cage in a crossover model. Their positions were reversed among replications. Approximately 200 mixed-sex horn flies of unknown age, starved 24 h before testing, were put inside the screen cage in a female:male ratio of 6:4, approximately, and the cage was covered with a perforated lid. Four hours in darkness were given to the flies for egg-laying to avoid phototaxic behaviors. Immediately after, the cages were frozen, and the flies were removed from the cages. The number of eggs on the treatments and the blanks was counted. The number of females in each screen cage was also recorded. The egg-laying on the treatments or on the blanks is expressed as number of eggs laid per female. This bioassay was replicated six times per sample. Differences in the number of eggs laid per female among treatments and blanks were analyzed using a generalized linear model (GLM) with a Poisson distribution and a log-link function, which is appropriate for count data. The analysis was performed using the statistical software Statistix 10 (Tallahassee, FL, USA). Model significance was assessed using the Likelihood Ratio Test. Post hoc comparisons between treatments were conducted by estimating predicted means and their confidence intervals, with non-overlapping intervals indicating significant differences.

### 2.5. Capture of Volatiles Emitted by Fresh and Aged Cattle Dung by SPME

Fecal samples (2 g per cow selected in Section 2.2) were placed in 30 mL vials sealed with a Teflon septum and screw cap. Volatiles emitted from fecal samples were captured by solid-phase microextraction (SPME) using the method reported by Tangtrakulwanich et al. [99], with modifications. Trapping was performed with a divinylbenzene/carboxene/polydimethylsiloxane (DVB/CAR/PDMS)-coated fiber (50/30 µm) (Supelco, Sigma-Aldrich, St Louis, MO, USA) and a manual SPME holder (Supelco, Sigma-Aldrich, St Louis, MO, USA). The SPME fiber was preconditioned for 30 min at the GC inlet at 250 °C with a continuous helium stream before volatile capture. The SPME fiber was exposed to volatiles from individual fecal samples for 24 h by manual insertion through the Teflon septum of the vial lid. Finally, the SPME fiber was inserted into the GC injection port for 1 min at 250 °C for volatile desorption.

### 2.6. Analysis of Dung Cattle Volatiles by GC/MS

Analysis of the volatile mixtures of the feces captured by SPME, as was indicated in Section 2.5, was performed by GC-MS using a Thermo Scientific TRACE 1300 Series gas–liquid chromatography (Waltham, MA, USA) coupled to a Thermo Scientific ISQ 7000 single quadrupole mass detector, with an integrated data system (Xcalibur 4.2.47, Thermo Fisher Scientific Inc., Waltham, MA, USA) and a 30 m long BPX5 capillary column (0.25 μm film thickness × 0.25 mm inner diameter, SGE Forte, Trajan Scientific and Medical, Ringwood, Victoria, Australia). Operating conditions were as follows. Injector, transfer line, and detector temperature: 250 °C; oven temperature program: 40 °C for 2 min, temperature increased to 250 °C at 5 °C min^−1^, and it was held at 250 °C for 5 min. He at 1 mL min^−1^ was used as carrier gas. The mass detector used an ionization energy of 70 eV. The recording conditions used a sweep time of 1.5 s and a mass range of 30 to 400 amu. Compounds were identified by comparison of their mass spectra with those in the NIST library 2.0 database (NIST, Gaithersburg, MD, USA), by comparison of their calculated retention index with those reported in literature [100] for the same type of stationary phase, and by co-injection of commercial standards. The calculated retention indices were determined by the retention time of *n*-alkane C8-26 standards (100 µg mL^−1^ in *n*-hexane) (Sigma-Aldrich, St. Louis, MO, USA) using the equation described by Kovats and Keulemans [101].

### 2.7. Ethical Approval

This study was evaluated and approved according to the record N° 041_22 provided by the Universidad de La Frontera ethics committee, Temuco, Chile.

## 3. Results

The volatile blends emitted from cattle dung samples were captured by SPME, and they were analyzed by GC-MS (Table 1). Accordingly, *p*-cresol (63.17%) was the most abundant compound in the volatile blend from fresh cattle dung, followed in lower amounts by phenol (0.84%); indole (5.46%); terpenes (9.73%), including *α*-pinene (1.05%), *β*-pinene (1.39%), limonene (1.52%), and *β*-caryophyllene (2.60%); and other common volatile organic compounds (VOCs) from cattle manure such as long-chain branched alcohols (0.65%), saturated branched hydrocarbons (1.41%), saturated unbranched hydrocarbons (3.28%), and unsaturated hydrocarbons (2.52%). Similar profiles were observed for volatile blends from aged cattle dung, which are compiled in Table 1.

**Figure 1:**
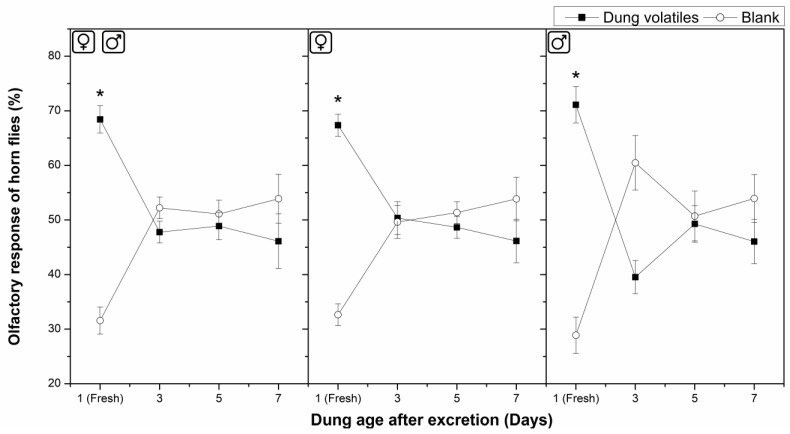
Olfactory responses of horn flies to volatiles from fresh and aged cattle dung. **Left**: Response of mixed-sex flies randomly selected; **Center**: response of female flies; and **Right**: response of male flies. Values are mean ± SD. Asterisks on the points indicate significant differences between treatments and blanks based on the Tukey HSD test (*p* ≤ 0.05); N = 6.

**Figure 2:**
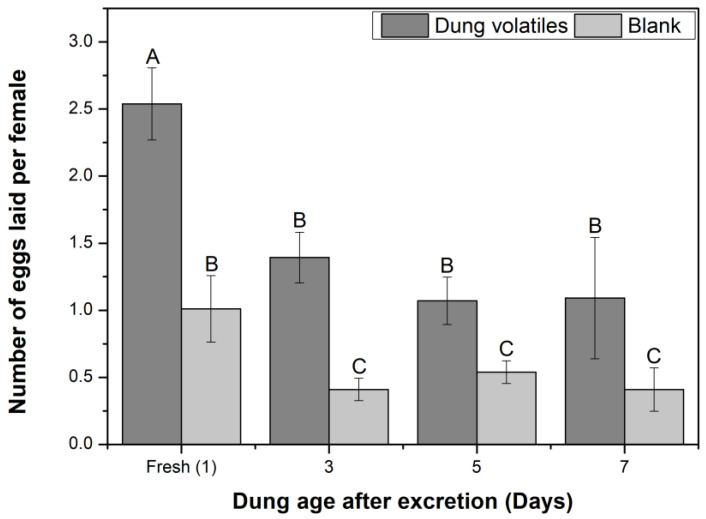
Oviposition of female horn flies exposed to volatiles from fresh and aged cattle dung. Columns are mean number of eggs laid per female horn fly ± SE. Different letters on the columns indicate significant differences among treatments, including blanks, based on the Likelihood Ratio (LR) test (*p* ≤ 0.05); N = 6.

Behavioral responses of horn flies to volatiles from fresh cattle dung (up to 24 h old) and 3–7-day-old cattle dung were investigated here (Figure 1 and Figure 2).

In the olfactory bioassays, volatiles from fresh dung significantly attracted (*p* ≤ 0.05) mixed-sex horn flies randomly selected, compared with the blank in a “Y”-tube olfactometer, with 68.4% ± 2.5% of attraction (Figure 1, Left). Contrary, volatiles from 3-, 5-, and 7-day-old dung did not cause a significant difference in the olfactory response of mixed-sex horn flies compared with their respective blanks, where 47.8% ± 2.0%, 48.9% ± 2.5%, and 46.1% ± 5.0% of the flies chose the treatment, respectively. Consequently, these samples were unattractive to mixed-sex horn flies (Figure 1, Left). In a similar manner, volatiles from fresh dung significantly attracted (*p* ≤ 0.05) males, with 71.1% ± 3.3% of attraction (Figure 1, Right), and females, with 67.4% ± 2.0% of attraction (Figure 1, Center), but again, volatiles from 3–7-day-old dung were unattractive to both horn fly sexes.

In the oviposition tests, egg number per female was significantly (*p* ≤ 0.05) higher in samples with cattle dung than in blanks, either in fresh dung or in aged dung (Figure 2). Comparing among treatments, the egg-laying was the highest in a sample with fresh cattle dung, with 2.54 ± 0.27 eggs per female, and it was significantly different from samples with 3-, 5-, and 7-day-old dung, where oviposition was 1.39 ± 0.19, 1.07 ± 0.18, and 1.09 ± 0.45 eggs per female, respectively. Egg-laying among samples with aged cattle dung was not significantly different. Considering the flies were separated from the feces with pieces of tulle, the egg-laying was influenced by volatile cues emanated from cattle feces.

**Figure 3:**
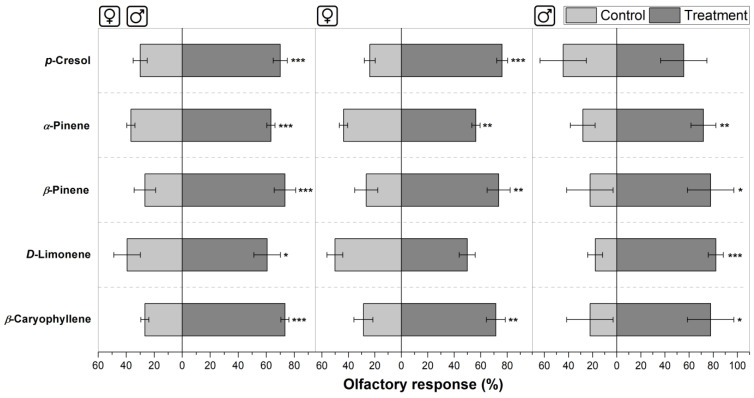
Olfactory responses of horn flies to single compounds present in volatile blend emanated from cattle dung. **Left**: Response of mixed-sex flies randomly selected; **Center**: response of female flies; and **Right**: response of male flies. Bars are mean ± SD. Asterisks next to the bars indicate significant differences between treatments and blanks based on the Tukey HSD test (* *p* ≤ 0.05, ** *p* ≤ 0.01, *** *p* ≤ 0.001); N = 6.

**Figure 4:**
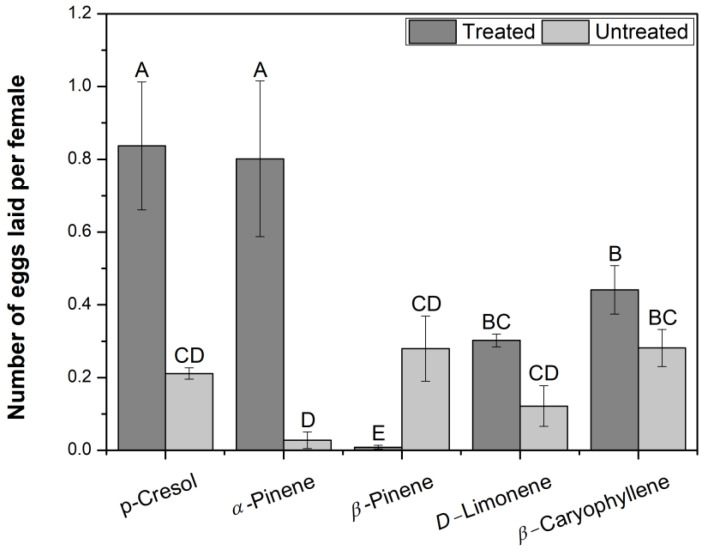
Oviposition of female horn flies exposed to single compounds present in volatile blend emanated from cattle dung. Columns are mean number of eggs laid per female horn fly ± SE. Different letters on the columns indicate significant differences among treatments, including blanks, based on the Likelihood Ratio (LR) test (*p* ≤ 0.05); N = 6.

Behavioral responses of horn flies to single compounds present in volatile blends emanated from cattle dung were also evaluated in this study (Figure 3 and Figure 4).

In the olfactory tests, the “Y”-tube olfactometer allowed us to measure the attraction provoked by most of the single compounds tested. *p*-Cresol, *α*-pinene, *β*-pinene, *D*-limonene, and *β*-caryophyllene at 0.05 µg per mL in hexane exhibited significant attraction in the olfactometer, compared with the blanks, where 70.0% ± 5.0% (*p* ≤ 0.001), 63.3% ± 2.9% (*p* ≤ 0.001), 73.3% ± 7.6% (*p* ≤ 0.001), 60.6% ± 9.5% (*p* ≤ 0.05), and 73.3% ± 2.9% (*p* ≤ 0.001) of the mixed-sex flies chose the treatment, respectively (Figure 3, Left). Testing with male flies, *α*-pinene, *β*-pinene, *D*-limonene, and *β*-caryophyllene were attractants, with 71.9% ± 10.3% (*p* ≤ 0.01), 77.8% ± 19.2% (*p* ≤ 0.05), 82.1% ± 6.2% (*p* ≤ 0.001), and 77.8% ± 19.2% (*p* ≤ 0.05) of male flies selecting the treatments, respectively. However, *p*-cresol did not cause a significant difference in the olfactory response of males compared with the blank, where 55.6% ± 19.2% of the flies chose the treatment (Figure 3, Right). Using female flies, *p*-cresol, *α*-pinene, *β*-pinene, and *β*-caryophyllene were attractive to females, where 76.2% ± 4.1% (*p* ≤ 0.001), 56.4% ± 3.1% (*p* ≤ 0.01), 73.5% ± 8.7% (*p* ≤ 0.01), and 71.4% ± 7.1% (*p* ≤ 0.01) of the flies chose the treatment. Differently, *D*-limonene was not attractive, where only 49.8% ± 6.0% of the female flies chose the treatment (Figure 3, Center). Therefore, *p*-cresol was unattractive to male horn flies, and *D*-limonene was not attractive to female horn flies.

In the oviposition bioassays, comparing among the single compounds, the egg-laying was the greatest in samples with *p*-cresol and *α*-pinene, with 0.84 ± 0.18 and 0.80 ± 0.21 eggs per female, respectively, and it was significant different (*p* ≤ 0.05) from its blanks, where the egg numbers per female were 0.21 ± 0.04 and 0.03 ± 0.01, respectively. Similarly, egg-laying in samples with *β*-pinene was significant different (*p* ≤ 0.05) from that in the blank, but in this case, the oviposition was higher in the blank than in the treatment, where each female laid 0.28 ± 0.09 eggs in the blank and 0.01 ± 0.005 eggs in the treatment. Samples with *D*-limonene and *β*-caryophyllene were not significantly different from the blanks. Considering the egg number laid per female in each treatment, it is possible to establish that *p*-cresol and *α*-pinene elicited a higher oviposition, *β*-pinene was an egg-laying deterrent, and *D*-limonene and *β*-caryophyllene were not active in the horn fly oviposition.

## 4. Discussion

The volatile blends emitted from cattle dung were principally constituted by *p*-cresol, followed to a lesser extent by phenol, indole, terpenes, long-chain branched alcohols, and branched and unbranched hydrocarbons, among others. Marked differences in volatile profiles from dung samples were not observed. However, taking in account the significant differences between the behavioral responses of horn flies exposed to volatiles from fresh and aged feces, further studies focused on the temporal variability in dung volatile emissions would be valuable. The compositions of dung blends observed here were representatives of common VOC emanations from the manure of grazing animals, including cows, horses, sheep, and goats [102]. In these volatile blends are various chemical groups of VOCs, including aromatic compounds (phenols and indoles), terpenes (isoprene, limonene, etc.), oxygenated compounds (e.g., cresol, acetaldehyde, etc.), sulfur compounds (methanethiol, dimethyl sulfide, etc.), and halogenated compounds, among others [102].

In the olfactory tests using a “Y”-tube olfactometer, volatiles from fresh dung significantly attracted mixed-sex horn flies, males and females. Moreover, in the oviposition tests, the egg number laid per female was always greater on the pieces of tulle covering cattle dung samples than that on blanks. Considering the flies were separated from the feces by pieces of tulle, the attraction and the egg-laying had to be elicited by volatile cues emanated from cattle feces, which passed across the tulle and were received by the flies. Additionally, volatiles from aged dung were neither attractive nor repellent to both horn fly sexes, and the egg-laying was minor over the aged dung, but it was not null. Therefore, fresh cattle dung was preferred by flies of both sexes and by female flies for egg-laying rather than aged dung. Nevertheless, the flies had the capability to select and to egg-lay on aged cattle dung anyway. It has been described in literature that most horn fly oviposition occurs in the first 2 min after excretion [1,15,103,104]; notwithstanding, these reports were based on detailed field observation, but the exact period of dung attraction has not been experimentally determined, until now. The results of this study are in agreement that the horn fly oviposition occurs mainly during the first minutes after excretion but also prove this period is not exclusive; in laboratory conditions, dung attraction to horn flies is conserved until 24 h after excretion, and the egg-laying may even occur in manure pats aged for several days. In relation with the olfactory responses of male horn flies, despite the attraction of male flies to volatiles from fresh dung that was observed here, it has not been documented in the literature if males are naturally interested in cattle manure pats in field. Nevertheless, the ecological approach taken to read these results should be weighed with special care regarding limitations inherent in the laboratory work. The olfactory bioassays did not consider visual and other non-visual cues. Moreover, the starved flies used in these analyses were constantly exposed to a light source during the olfactory tests. These conditions do not exactly represent natural conditions in which wild horn flies develop their life cycle. Thus, field studies would be valuable to complement these findings.

This study demonstrates, for the first time, the role of volatile semiochemicals emanated from cattle dung in conferring, in part, the attractiveness of it to horn flies, and in stimulating the oviposition of female horn flies, without considering visual and other non-visual cues. Notwithstanding the aforementioned, there is not more information in the literature about the role of semiochemicals emitted from cattle dung in dung selection and in the oviposition of the *H. irritans*. However, it has been reported that horse and cow manure were attractants to stable flies, especially females searching for oviposition sites [97,98]. When stable flies were offered a choice between these two oviposition substrates, the flies always chose horse dung over cow dung. However, analyses of volatiles emanating from horse and cow dung by coupled gas chromatography–electroantennography (GC–EAG) with stable flies revealed no differences in the chemical cues released from both substrates. The major EAG active compounds in both substrates were butanoic acid, 1-octen-3-ol, decanal, octan-3-one, skatole, dimethyl trisulfide, *β*-caryophyllene, and *p*-cresol [97]. Behavioral responses of stable flies to volatile cues emanating from manure slurry have also been investigated [99]. *p*-Cresol, *m*-cresol, and phenol, among others, were detected by GC-MS of SPME captures from cattle manure slurry. Olfactometer assays showed that *p*-cresol, *m*-cresol, and phenol at the lowest dosages (10 μL of 0.1 μg μL^−1^ solutions) elicited significant attraction, but at higher dosages, they appeared to be unattractive and even acted as repellents for stable flies. Mixtures of phenol and either *m*-cresol or *p*-cresol (10 μL) at 10 μg μL^−1^ were attractive to stable flies. Additionally, traps placed in fields with an application of manure slurry caught over 10 times more flies than those placed away from sprayed sites [99].

In this study, five single compounds identified in volatile blends from cattle manure were selected to evaluate their effects on the olfactory response and oviposition of horn flies. Among the selected compounds, *p*-cresol was chosen due to it being the major component in the volatile blend from fresh cattle dung. Furthermore, it provoked significant EAG responses in antennae from stable flies [97,99] and horn flies [21,87], and it elicited the attraction of stable flies [99] and un-sexed horn flies [21,87]. The sesquiterpene *β*-caryophyllene was chosen in the current study, considering its significant EAG activity provoked in *S. calcitrans* antennae. The monoterpene *D*-limonene, also known as *R*-(+)-limonene, was chosen taking into account the effects on *H. irritans* informed by Showler et al. [64]. Moreover, considering the well-known anti-insect properties of terpenes [105], *α*-pinene and *β*-pinene were also chosen, although these compounds were minor components in the volatile blends from cattle feces. To minimize the amount of chemical cues to apply, single compounds were tested using 10 μL of 0.05 μg mL^−1^ in *n*-hexane solutions. This concentration was established at half that of the *p*-cresol concentration, tested by Oyarzún et al. [21], Birkett et al. [87], and Tangtrakulwanich et al. [99], on stable flies or horn flies (10 μL of 0.1 μg μL^−1^ solutions).

*β*-Caryophyllene, which was EAG-active in *S. calcitrans* antennae [97], and *p*-cresol, which was EAG-active and attractive for stable flies [97,99], acted as semiochemicals for horn flies in the present study but using half of the concentration used for stable flies. Here, *β*-caryophyllene was attractive to horn flies of both sexes, but it was not active in the egg-laying behavior. *p*-Cresol was attractive to mixed-sex flies, and it was also an attractant for females. Moreover, it elicited the oviposition. Related with the egg-laying bioassays using the chosen compounds, it is important to mention that aged feces were used as oviposition substrate, separating flies and dung with pieces of tulle; therefore, the egg-laying was influenced by the addition of specific compounds to the aged cattle dung.

As mentioned above, the EAG and olfactory responses of horn flies to *p*-cresol were reported previously [21,87]. Multiple active peaks in volatile extracts collected from the bodies of Holstein–Friesian heifers were recorded by GC–EAG using *H. irritans* antennae. These compounds included *p*-cresol [87] and others previously reported to influence tsetse fly behavior in field [89,106,107]. Another study provided evidence that individual Holstein–Friesian heifers were differentially attractive to *H. irritans* [21]. GC-MS analysis of volatiles collected from heifers revealed the presence of compounds previously reported as semiochemicals for cattle flies [87,94,95,107,108], including *p*-cresol and *m*-cresol. In Y-tube olfactometer tests, both *p*-cresol and *m*-cresol attracted horn flies at the highest concentrations tested (10 μL of 0.1 μg μL^−1^ solutions), which is consistent with the attraction observed in the present study but here used just half of that concentration. Surprisingly, volatile blends collected from high and low fly-load heifers were not attractive in the previous work [21]. However, the sex of the flies was not considered.

The behavioral responses of horn flies to *α*-pinene, *β*-pinene, *β*-caryophyllene, and *D*-limonene were also tested at the current study. It is noteworthy to mention that racemic mixtures of *α*-pinene, *β*-pinene, and *β*-caryophyllene and the (*R*)-enantiomer of limonene have been used in this study. However, the absolute configuration of the terpenes emitted from cattle dung was not determinate. Thus, there might be mismatch between the enantiomeric composition of terpenes released from the dung samples and those of commercial samples tested for bioactivity. Nonetheless, all of these terpenes were attractive to both sexes, male and female horn flies, with the exception of *D*-limonene, which was unattractive to females. The egg-laying also was influenced by the single terpenes added on pieces of tulle covering dung. *α*-Pinene elicited the oviposition of the female flies, while *β*-pinene acted as an egg-laying deterrent, and *D*-limonene and *β*-caryophyllene were not active. The olfactory responses of horn flies to *D*-limonene (*R*-(+)-limonene) have also been reported previously [64]. When *D*-limonene and a limonene-based insecticide (Orange Guard, Carmel Valley, CA, USA) containing 5.8% *D*-limonene steam distilled from citrus peels were tested, 74% to 90% of the horn flies were attracted to all concentrations of Orange Guard and the laboratory-grade limonene tested [64]. Although the sex of the flies was not considered, and concentrations were much higher than those tested in this study, this is in accordance with the attraction to horn flies observed here.

The observed attraction of female flies to *β*-pinene, despite it provoking reduced egg-laying on dung treated with this compound, is an intriguing result. There is outdated information concerning the *H. irritans* ecology in the literature [3,8,14,15,79,80,81]. Twenty-five years ago, Kuramochi [15] observed in field that female horn flies obtained blood meals from the bovine host before oviposition. This would therefore suggest that female flies were attracted to *β*-pinene as a feeding signal. However, the feeding activity of *β*-pinene on *H. irritans* has not been determined. Another plausible explanation is concerning to the effects of the particular compound compared with the effects of compounds in a complex mixture. In this scenario, Picket et al. [81] reported that mixtures of 3-*n*-propylphenol, 1-octen-3-ol, *p*-cresol, and acetone were partially attractive to *Glossina* spp. populations compared to natural cattle host odor, implying that other attractants need to be identified in order to provide full activity. Additionally, it is well-known that the insect responses are often concentration-dependent. For example, Birkett et al. [87] proved that single compounds that showed EAG activity for cattle fly species provoked different responses in a wind-tunnel bioassay with face flies, *Musca autumnalis* (de Geer) (Diptera: Muscidae). The 1-octen-3-ol, 6-methyl-5-hepten-2-one, and 3-octanol showed significant attraction at certain concentrations, with 1-octen-3-ol attracting flies at very low levels. Naphthalene and linalool showed strong repellency at low concentrations, but at higher concentrations, responses were not significantly different from the those for the control. Therefore, more studies are necessary to evaluate the effects of the compound concentration and blends on the *H. irritans* behavior.

It should be highlighted that although the attraction of *p*-cresol [21], *D*-limonene [64], and the effects of some essential oils [109] on horn flies have been previously described, this is the first report of the effects of *p*-cresol, *α*-pinene, *β*-pinene, *β*-caryophyllene, and *D*-limonene on the horn fly oviposition and on the olfactory response differentiating by sex. The results of this study revealed that *p*-cresol and *α*-pinene may be responsible, in part, for the dung selection by female horn flies searching for oviposition sites. Moreover, the olfactory responses of horn flies to volatile cues were dependent on the *H. irritans’* sex. Therefore, in this kind of behavioral test, recognizing the fly sex becomes extremely important, considering the life cycle of the *H. irritans*, where males and females show different mating and egg-laying behaviors, among others [1].

## 5. Conclusions

In this research, the effects of volatile blends and individual compounds emitted from fresh and aged cattle dung on the olfactory response of horn flies and on the oviposition of female horn flies were successfully evaluated. The olfactory response and the egg-laying of horn flies were influenced by volatile cues emanated from cattle feces. Volatile cues from fresh cattle dung were preferred by flies of both sexes and by female flies for egg-laying rather than aged dung. Moreover, *p*-cresol and *α*-pinene were attractive to female horn flies, and they elicited a higher oviposition. Nonetheless, *p*-cresol was unattractive to males. Contrarily, *β*-pinene was an egg-laying deterrent, and *β*-caryophyllene was not influential on the oviposition, although these terpenes were attractive to both sexes of flies. *D*-Limonene was not active in the horn fly egg-laying and was also unattractive to female flies. Therefore, *p*-cresol and *α*-pinene may be responsible, in part, for the dung selection by female horn flies searching for oviposition sites. However, more single semiochemicals and mixtures of these, in different concentrations, should be tested on the olfactory and oviposition behavior of horn flies. Additionally, more studies are necessary to establish significant differences between the volatile profiles from cattle dung.

The role of volatile cues emanated from cattle dung involved in the dung selection and oviposition of horn flies had been unexplored, until now. Principally, studies with host and dung semiochemicals were centered in stable fly behaviors. Moreover, the behavioral responses of horn flies in laboratory and in field have been tested to very few semiochemicals from cattle hosts or cattle dung. The use of semiochemicals has demonstrated being a promising tool in stable fly management. Stable fly trap innovations have included the addition of host-associated attractive odorants, principally phenol and *m*-cresol, to increase capture rates. However, innovative trapping technologies to catch horn flies have been unexplored. Thus, the present research could contribute to the development of new approaches for the control of the horn fly using semiochemicals as lures in fly traps and simultaneously emitting repellents from cattle, as part of a “push-pull” strategy.

## Figures and Tables

**Table 1:** Chemical composition of the volatile blend emitted from fresh and aged cattle dung.

RT	KI	Compound	Fresh Dung	Aged Dung *	Identification
			Abundance (%)	Abundance (%)	
11.9	932	*α*-Pinene	1.05	0.02–0.10	RI, MS, Co-I
13.3	966	*β*-Pinene	1.39	0.27–0.32	RI, MS, Co-I
14.5	1000	Decane	1.22	0.84–1.55	RI, MS
15.3	1022	Limonene	1.52	0.11–0.16	RI, MS, Co-I
16.5	1061	Undecane	2.06	0.05–0.24	RI, MS
16.8	1071	Phenol	0.84	2.14–39.63	RI, MS, Co-I
17.3	1086	*p*-Cresol	63.17	26.48–43.02	RI, MS, Co-I
23.5	1295	1-Tridecene	0.44	1.00–1.56	RI, MS
25.1	1358	Indole	5.46	1.03–2.95	RI, MS, Co-I
26.3	1399	Isocaryophyllene	2.35	1.37–2.16	RI, MS
26.5	1411	*β*-Caryophyllene	2.60	1.66–3.24	RI, MS, Co-I
27.7	1458	2,6,10-Trimethyltridecane	0.81	0.49–0.91	RI, MS
28.3	1480	Guaia-6,9-diene	0.82	0.37–0.46	RI, MS
32.4	1660	*cis*-9-Tetradecen-1-ol	0.65	0.18–8.76	RI, MS
35.2	1789	1-Heptadecene	1.62	1.69–5.03	RI, MS
35.7	1813	2,6,10,14-Tetramethylhexadecane	0.60	0.36–0.73	RI, MS
36.4	1848	*trans*-Phyt-2-ene	0.46	0.84–2.13	RI, MS
		Unknown compounds	12.94	9.36–32.65	

RT: retention time (min), RI: Kovats retention index, %: considering detected compounds, Co-I: co-injection, * lower and upper values of abundance for blends of aged cattle dung.

## Data Availability

Data are contained within the article.
